# In vitro assessments of nanoplexes of polyethylenimine-coated graphene oxide-plasmid through various cancer cell lines and primary mesenchymal stem cells

**DOI:** 10.1371/journal.pone.0295822

**Published:** 2023-12-14

**Authors:** Parichehr Maleki, Ali Dinari, Babak Jahangiri, Jamshid Raheb

**Affiliations:** 1 Department of Molecular Medicine, Institute of Medical Biotechnology, National Institute of Genetic Engineering and Biotechnology, Tehran, Iran; 2 Research Center for Nanorobotics in Brain, Gwangju Institute of Science and Technology (GIST), Gwangju, South Korea; Brandeis University, UNITED STATES

## Abstract

Efficient gene therapy relies on an efficient gene delivery system. Viral gene delivery approaches excel in transferring and expressing external genes; however, their immunogenicity and difficulty in large-scale production limit their clinical applications. In contrast, nanoparticle-based gene delivery systems have gained increasing attention due to less immunogenicity and more convenience for large-scale production. Nevertheless, their poor transfection efficiency compared to viral systems remains a significant obstacle. In the present study, we investigated the transfection efficiency of our PEI-coated graphene oxides in HEK293T, Calu-3, Calu-6 cell lines, and primary human bone marrow mesenchymal stem cell (MSC). The high surface ratio and good biocompatibility of graphene oxide make it an appealing tool for gene delivery systems. However, the low dispersity of graphene oxide in aqueous environments is the first barrier that needs to be conquered. For this, we enhanced the dispersity and stability of graphene oxide in water by sonicating it for at least 5 hours at a pH of 7. Then, graphene oxide was conjugated with branched PEI (25 kDa) to have a positive charge, enabling it to condense nucleic acids with a naturally negative potential. The physio-chemical characteristics of our synthesized nano-carriers (GO-PEI) were determined by DLS, FT-IR, and AFM. The utilized plasmid in polyplexes contained a GFP gene, allowing us to verify transfection efficiency through fluorescent microscopy and flow cytometry. While GO-PEI carriers were highly efficient in transfecting HEK293T cells, the transfection efficiency in MSCs and Calu-3 cells was notably low. We suppose that the main reason for the low transfection efficiency of GO-PEI in these cells is due to its higher toxicity. Despite this, considering the various advantages of graphene oxide in drug delivery as well as its optical and electrical applications in biomedicine, we propose to functionalize graphene oxide with more biocompatible materials to enhance its potential as a successful gene carrier in these cell types.

## 1. Introduction

Gene therapy has been noticed as a revolutionary approach in treating of incurable disorders by utilizing genetic materials to manipulate gene expressions. An appropriate gene delivery system is the first requirement for successful gene therapy. In gene therapy, genetic materials such as plasmid DNAs, small interfering RNAs (siRNAs), short hairpin RNAs (shRNAs), and the CRISPR/Cas9 system need to be safely transferred to a targeted tissue with negligible toxicity to other tissues. Efficient transfer of these materials needs a proficient delivery vehicle to properly penetrate the cell membranes, either in vitro or in vivo. Moreover, an efficient delivery system is necessary to protect genetic materials from digesting enzymes, immune system attacks, high renal clearance, and escape from the endosomal system [1]. The gene delivery systems are divided into two main categories: viral and non-viral vectors. Currently, viral vectors are the most utilized vectors on the market. The superior advantage of viral vectors is their higher rates of transfection efficiency. However, there are some drawbacks to the viral vectors, such as immunogenicity, safety hazards, the integrity of the viral genome in the host’s genome, and high production costs, which limit their application. On the other hand, non-viral vectors are usually more attractive due to their low immunogenicity, ability to load larger amounts of cargo, high safety, low cost, and ease of production on large scales. Providing all these features simultaneously into a single carrier is challenging, particularly as some of them are contradictory. For example, carriers with high transfection rates usually indicate lower biocompatibility [2–4]. Graphene-oxide (GO) is a desirable material to use as a nano-carrier among various types of nanoparticles owing to its large surface ratio, good biocompatibility, strong mechanical properties, aqueous colloidal stability, and great electrical conductivity [5–8]. Graphene has a monolayer structure that makes every atom exposed on its surface, which provides a bigger space for loading various cargoes [9]. The existence of multiple reactive groups, like hydroxyl groups, on the surface of GO facilitates its conjugation with ligands. Another attraction of GO for biomedical applications is its good biocompatibility because of its myriad hydrophilic groups. Smart-bio imaging is another desired application of GO due to its intrinsic optical features, which is an advantage for using it in drug delivery approaches [10, 11]. Another advantage of GO is its cost-effective and convenient large-scale production. However, both GO and genetic materials have negative charges, posing a challenge for the efficient loading of genetic derivatives onto the GO carriers. To overcome this obstacle, covalent modification of GO is a traditional approach, which not only enhances the transfection efficiency but also improves GO stability and reduces its cytotoxicity. In this regard, The conjugation of polymers like PEI [12], PEG [12–14], and chitosan [15–17] are the most commonly applied polymers for the functionalization of GO for gene delivery. PEI, a prevalently utilized reagent for gene transfection, is a cationic polymer with primary, secondary, and tertiary amino groups with excellent water solubility. The presence of high amine density makes PEI a promising reagent for efficient transfection [18]. Therefore, coating GO with PEI is beneficial to achieve an efficient nano-carrier (GO-PEI) by combining the appealing features of both GO and PEI. For instance, GO-PEI is less cytotoxic compared with PEI [19], probably due to the greater biocompatibility of GO than PEI. On the other hand, conjugation of PEI on the surface of GO gives it a positive charge, enabling GO to condense negatively charged cargoes like genetic materials. Several studies have explored the concept of nano-carriers composed of GO and PEI, yielding diverse outcomes, likely contingent on the specific cell types involved. For instance, Chen et al. synthesized GO-PEI carriers with branched PEI 25 kDa, with an appropriate transferring ability of plasmids to HeLa cells. Their observed transfection efficiency was comparable to or even higher than transfection with bare PEI 25 kDa [19]. Kim et al. synthesized an efficient GO-PEI construct with branched PEI 1.8 kDa for transferring siRNAs into the Hela and PC-3 cell lines, aligning with the purpose of functioning as a bio-imaging tool [20]. Ren et al. equipped their GO-PEI with a nuclear-localized signal, PV7 peptide, to enhance the expression of the transfected gene in HEK 293T and Hela cell lines [21].

## 2. Materials and methods

### 2.1. Materials

The graphene oxide powder (GO) was purchased from Nanosany Corporation company, Iran. Branched polyethylenimine (bPEI; molecular masses of 25 kDa), Triethylamine (TEA), 1-Ethyl-3-(3-dimethylaminopropyl) carbodiimide hydrochloride (EDC), N-Hydroxysuccinimide (NHS), and methylthiazole tetrazolium [3-(4, 5-dimethylthiazol-2-yl)-2, 5-diphenyltetrazolium bromide] (MTT; tissue culture grade) were obtained from Sigma.

### 2.2. Synthesis of conjugated GO-PEI

We established a stable graphene oxide system in an aqueous environment before the conjugation of PEI onto the surface of graphene oxide. To achieve this, 1 mg of GO was dispersed in each 1 ml of deionized water and homogenized at 20.000 spin/min using a Miccra-model D-1 –homogenizer or sonicated with a probe ultrasound sonicator in 50% amplitude (Hielscher ultrasound technology with 50 to 400 W), separately in different time periods at a pH of 7 to 7.5 to attain a stable and dispersed graphene oxide aqueous system. The most stable system earned from the previous step was used for conjugation with bPEI 25kDa through EDC/NHS coupling. Briefly, 5 ml of GO (1 mg/ml) underwent sonication for 30 minutes in the ultrasonic bath and was subsequently filtered using a 400 nm polycarbonate syringe filter. Simultaneously, 5 ml of PEI (10 mg/ml) underwent sonication in the ultrasonic bath, followed by mixing with 500 μl TEA. The obtained PEI-TEA solution was then added to the GO. EDC and NHS were prepared in concentrations of 10 mg/ml each and were added to the GO and PEI in two steps. Initially, 2 ml of each EDC and NHS solution were combined with GO and PEI on a stirrer for 30 minutes. In the second step, the rest of the EDC and NHS solutions were introduced to the reaction, and left on the stirrer for 24 hours at room temperature in a dark condition (the pH of the reaction was set at 8–8.5). The resulting GO-PEI solution was dialyzed with a 10 kDa molecular mass cutoff dialysis membranes in deionized water for three days to eliminate the unbound PEI from the final GO-PEI solution.

### 2.3. Physical characterization

#### 2.3.1. FT-IR spectroscopy

FT-IR was applied to validate the successful binding of PEI on the surface of graphene oxides. For this, 1 mg of dried samples were used to prepare KBr pellets for each sample. Then, FT-IR spectra were recorded on a Bruker Tensor 27 FT-IR spectrometer.

#### 2.3.2. DLS analysis

The hydrodynamic size and zeta potential of nano-carriers and polyplexes were measured using dynamic light scattering (DLS) with a Malvern Zetasizer device.

#### 2.3.3. Atomic Force Microscopy (AFM)

The size and morphology of GO and GO-PEI were determined using atomic force microscopy with an NT-MDT AFM NT-MDT AFM apparatus in noncontact mode, utilizing an NSG 10 tip with a resonance frequency of 190 kHz. A droplet of each sample (50 μg/ml) was applied onto a fresh silicon wafer and subsequently dried at 80°C. The taken images were analyzed using Image J software.

### 2.4. Toxicity assay

The MTT assay was performed to evaluate the toxicity effect of GO-PEI on the cell viability at 24 and 48 hours post-treatment. Briefly, 1–3 × 10^4^ cells (depending on the specific cell line) were seeded per well in 96-well plates and treated with various concentrations of GO-PEI or PEI. After 24 and 48 hours, 10 μl of MTT solution (5 mg/ml) was added to each well containing 100 μl media and incubated for an additional four hours. Then, to dissolve the formazan crystals, the media were replaced with 50 μl dimethyl sulfoxide (DMSO, Sigma-Aldrich). Absorbance data were then measured using a microplate photometer (LabSystem Multiscan) to calculate the cell viability affected by the treatments.

### 2.5. Gel retardation assay

Nucleic acids (plasmid and siRNA) were loaded on GO-PEI and PEI to prepare polyplexes at different N/P ratios, which were calculated according to the Formula (1) introduced by Cheraghi et al. [22]. N/P represents the molecular ratio of amine groups of PEI to phosphate groups of nucleic acids. The employed amounts of nanoparticle (N) and nucleic acids (P) were respectively variable and constant (= 1μg) among all samples (N/Ps). Therefore, the determined concentrations of nanoparticles were added to DNA solutions, resulting in a final volume of 50 μl for each mixture. The mixtures were then incubated at room temperature for 30 minutes. Subsequently, the polyplexes were loaded onto a 1% agarose gel containing 1X TAE buffer and electrophoresed for 1 hour at 80 V, followed by staining with ethidium bromide. Then, the gel was visualized with a UV gel documentation system (UV Gel Doc).


$$\frac{N}{P}=\frac{\frac{\frac{weight\;of\;nanocarrier(\mu g)}{molecular\;weight\;of\;nanocarrier}}{number\;of\;positive\;charge}}{\frac{weight\;of\;DNA\;(\mu g)}{mean\;of\;molecula\;weight\;of\;dNMPs}}$$ (1)

### 2.6. Cell culture and transfection

Calu-3, Calu-6, and HEK293T cell lines were obtained from the Pasture Institute of Iran. Primary MSC (human bone marrow- mesenchymal stem cells) was purchased from the Rouyan Institute of Iran. All the examined cancer cell lines were cultured in high-glucose Dulbecco’s Modified Eagle’s Medium (DMEM, Gibco) containing 10% fetal bovine serum (FBS, Gibco) and 1% penicillin-streptomycin. MSCs were cultured in low-glucose DMEM supplemented with 1% non-essential amino acids, 1% Glutamax, 10% FBS, and 1% penicillin-streptomycin. Cells were kept in an incubator (Binder) with 5% CO2 and 98% humidity at 37°C. For all of the transfections, 1000 ng of DNA was applied in the present work. Various N/P ratios were used for transfection with GO-PEI and PEI polyplexes. Transfections with Lipofectamine 2000 were performed according to the manufacturer’s instructions as a control in the current work. Typically, a day before transfection, the optimal number of cells were seeded in each well of a 24-well plate. During transfection, the confluency of cells was nearly 70%, and the cells were serum-starved and antibiotic-free. Media replacement with complete medium occurred 4 hours after transfections. Finally, the efficiency of transfections was evaluated through observation under a fluorescent microscope and quantification with a flow cytometry device. For quantification of transfection, GFP expressing cells were marked.

### 2.7. RNA extraction, cDNA synthesis, and real-time PCR

The total cellular RNA was extracted using TriPure Isolation Reagent (Roche, Germany) according to the manufacturer’s instructions 48 hours after transfections. Then, cDNA was synthesized by RevertAid First Strand cDNA Synthesis Kit (Thermo Fisher Scientific, Inc) utilizing 2 μg of total extracted RNA previously treated by DNaseI (Thermo Fisher Scientific, Inc). Finally, the real-time polymerase chain reaction (PCR) was performed using a SYBR Green PCR kit (Roche) in the Magnetic Induction Cycler system for the quantitative expression analysis of the targeted gene. It’s worth mentioning that the accuracy of the detected *LINK-A* PCR product was confirmed in our previous study [23].

## 3. Results

### 3.1. Fabrication of PEI–coated graphene oxides

**Fig 1:**
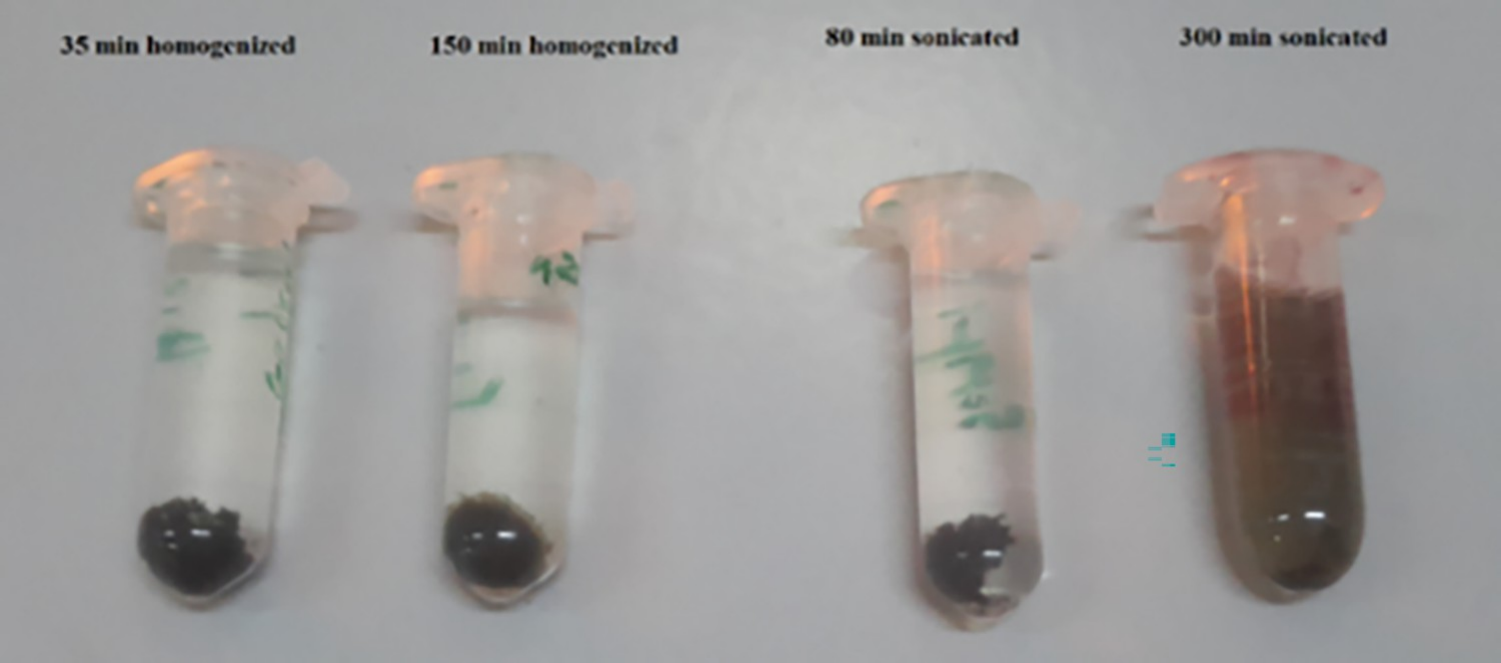
Dispersity of aqueous graphene oxide systems with various durations of sonication or homogenization after six months.

The low dispersity of graphene oxide in the aqueous environment was the first barrier facing its application for biological purposes. To address this, we employed distinct methods of long-term sonication and homogenization separately to provide a stable graphene oxide system. Sonication, performed with a probe sonicator at approximately 50% amplitude for varying durations (40, 80, and 300 minutes), and homogenization at 1400 rpm for different intervals (15, 35, and 150 minutes) were utilized. Both sonication and homogenization were expected to increase the dispersity of graphene oxide by exfoliating its layers. Moreover, pH is an imperative factor in maintaining the stability of aqueous graphene oxide systems. Although pH ranges from 3 to 11 are generally suitable for graphene oxides in water, optimal pH varies for different-sized particles due to the distribution of carboxylic groups on their surfaces, which are highly sensitive to acidic and alkaline environments [24]. We observed that our graphene oxide system had the best stability and dispersion at pH 7 to 7.5. DLS data indicated that the sonicated graphene oxide for 300 minutes exhibited the most negative surface charge (-45.1 mV) among the other treated graphene oxides, representing the most stable form of graphene oxide in water. This sonicated graphene oxide remained stable at room temperature for over six months, while homogenized samples precipitated in less than one hour, as shown in Fig 1. Subsequently, PEI was conjugated to the surface of this stabilized graphene oxide using EDC and NHS.

### 3.2. Physicochemical characteristics of graphene oxides and PEI–coated graphene oxides

#### 3.2.1. Zeta potential

**Fig 2:**
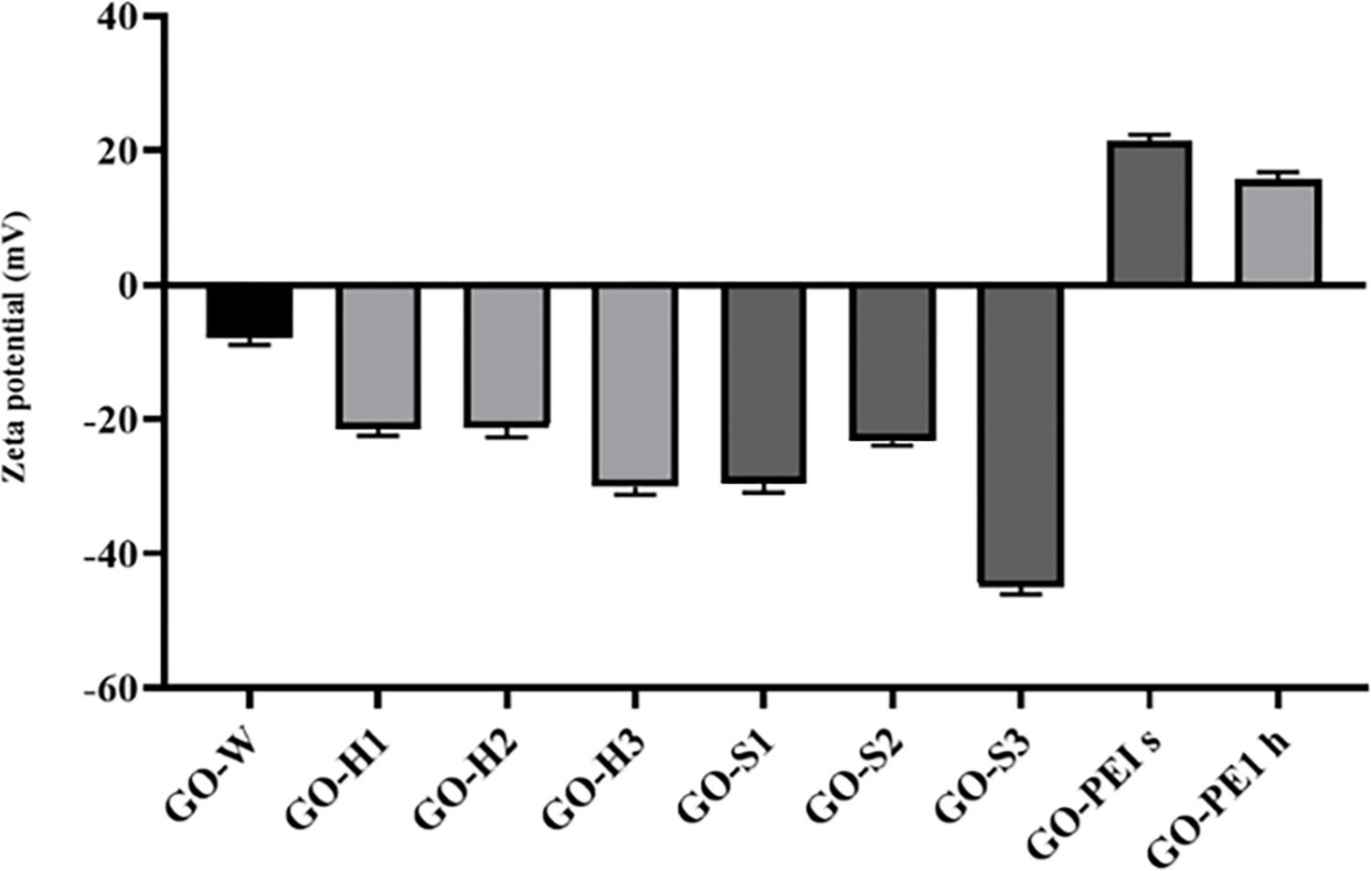
Zeta potential of graphene oxides. GO-W: Graphene oxide without any treatment, GO-H1: 15 minutes homogenized graphene oxide, GO-H2: 40 minutes homogenized graphene oxide, GO-H3: 35 minutes homogenized graphene oxide, GO-S1: 80 minutes sonicated graphene oxide, GO-S2: 150 minutes sonicated graphene oxide, GO-S3: 300 minutes sonicated graphene oxide, GO-PEI s: sonicated graphene oxide conjugated with PEI, GO-PEI h: homogenized graphene oxide with PEI. The data are presented with mean ± SD.

DLS was employed to assess the zeta potential or surface charges of the nano-carriers. It is anticipated that well-exfoliated graphene oxides would exhibit more negative surface charges because of the increased exposure of functional groups to water molecules. The zeta potential of the purchased graphene oxide, without any treatment, was recorded at -7.86 mV, which was the less stable form of graphene oxide in water. However, following sonication and homogenization, there was a notable increase in the negative surface charge (Fig 2). Our observations indicated a correlation between a negative zeta potential and the stability or effective dispersity of graphene oxide in water. Consequently, we selected graphene oxide with a -45.1 mV zeta potential for conjugation with PEI, intending to employ it in transfections for this study. As explained before, the sonicated graphene oxide for 300 minutes exhibited the best dispersity in water, even after a year. The Zeta potential of GO-PEI was changed to positive values, affirming the successful bonding of PEI onto graphene oxides.

#### 3.2.2. FT-IR results

**Fig 3:**
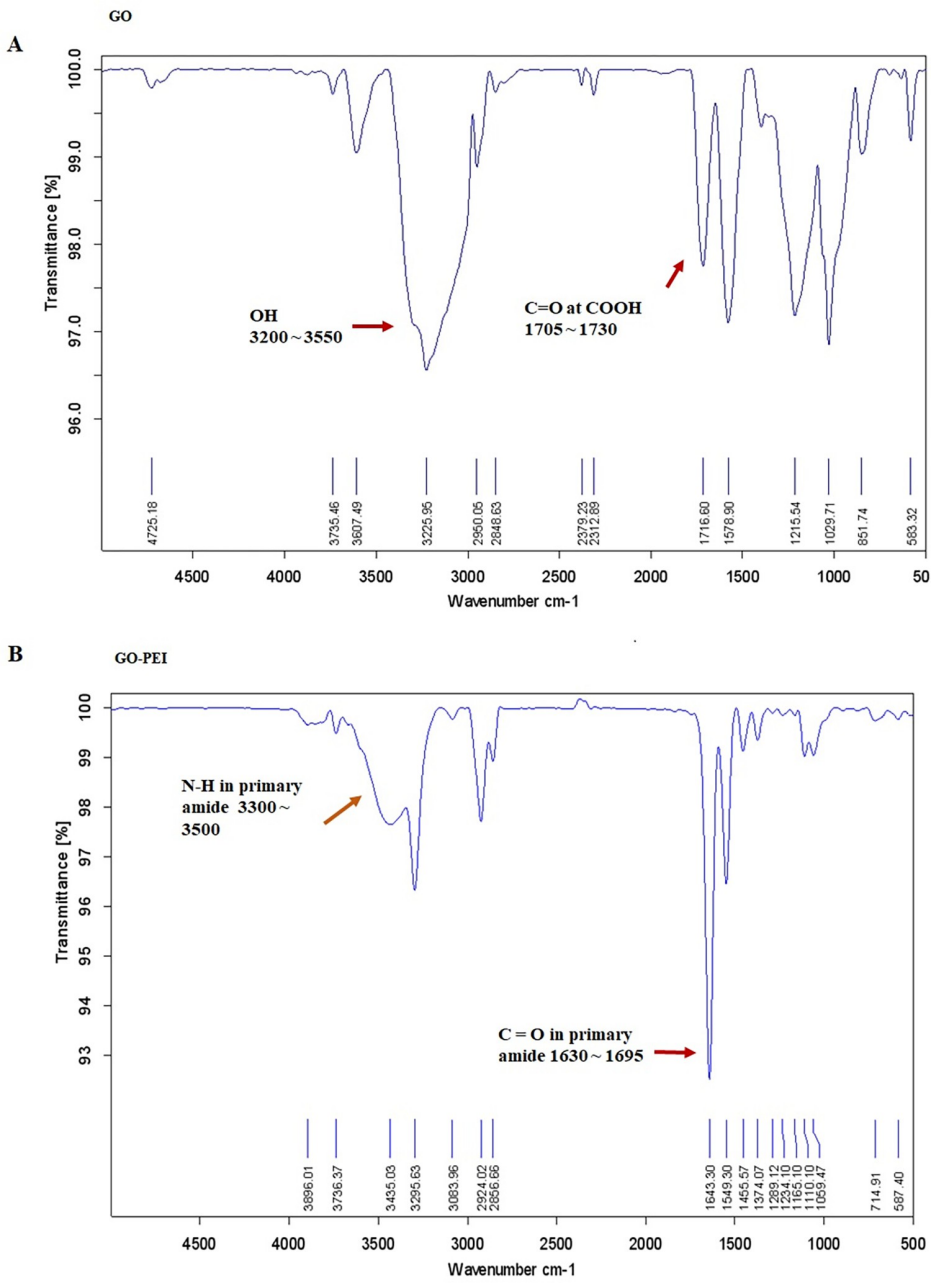
FT-IR graphs. (A) FT-IR graph of graphene oxide. (B) FT-IR of PEI conjugated graphene oxide. The disappearance and appearance of the indicated peaks in graphene oxide (GO) and graphene oxide-polyethylenimine (GO-PEI), respectively, prove the binding between GO and GO-PEI.

Data obtained from FT-IR also proved the successful conjugation of PEI to graphene oxides. As mentioned before, the positive zeta potential of GO-PEI is an indicator of the binding between PEI and graphene oxide. The appearance of the amide C = O bond peak at 1630–1695 cm^-1^ and the disappearance of the carboxylic group bond’s spike at 1705–1730 cm^-1^ in GO-PEI signify the effective conjugation of PEI and graphene oxide. Moreover, the presence of the absorption band corresponding to N-H stretches at 3300–3500 cm^-1^ in GO-PEI instead of OH peak at 3200–3550 cm^-1^ in GO further supports the successful production of GO-PEI (Fig 3).

#### 3.2.3. Measuring the size of nanoparticles

**Fig 4:**
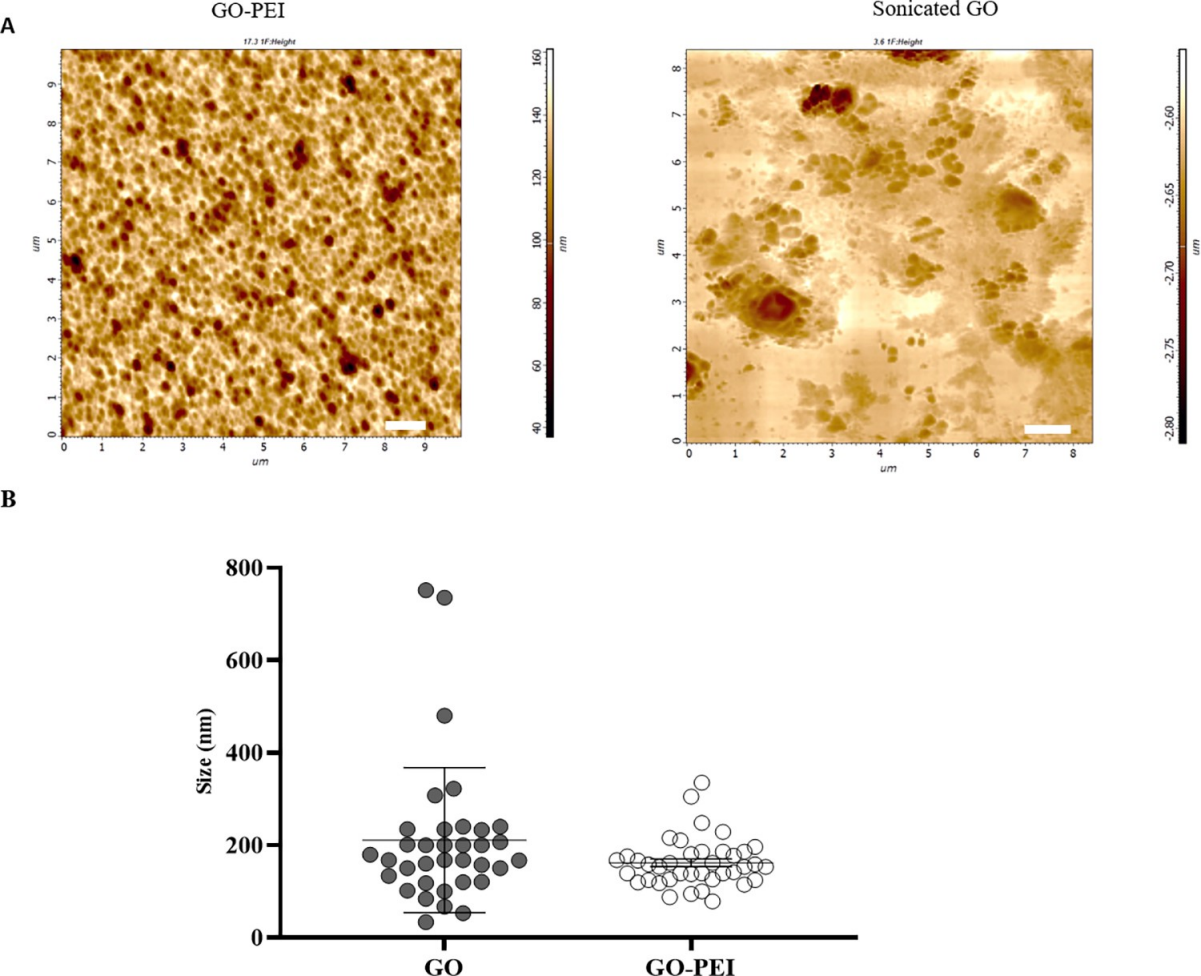
Size measurements of nanoparticles by AFM. (A) Images of sonicated GO and fabricated GO-PEI taken by AFM, the white line indicates 1μm in each image. (B) Scatter plot of size distribution for GO and GO-PEI, the number of particles was counted using Image J analysis. The data are presented with mean ± SEM.

Atomic force microscopy (AFM) was used for nanoparticle size assessment. Image J analysis of AFM-captured nanoparticle images revealed average sizes of 227.06 nm for sonicated graphene oxide and 161.61 nm for PEI-coated sonicated graphene oxide (Fig 4). The smaller size of GO-PEI compared with bare graphene oxides could be attributed to the conformational changes and the bending of PEI conjugated graphene oxides at the edges [25]. This conformational change can be explained by formation of an amide bond with the carboxylic groups located at the edges of graphene oxide sheets and a ring-opening reaction with the epoxy groups at the basal plane of the graphene oxide, facilitated by the primary amines of PEI [20, 25]. Moreover, the binding of PEI to graphene oxides resulted in a more uniformly dispersed distribution of GO-PEI particles compared to GO particles, as depicted in Fig 4. This improved distribution arises from the conjugation of PEI at the edges, which exerts an inhibitory effect on the accumulation of multiple layers of graphene oxide sheets and prevents their edge-to-edge arrangement through their functional groups [26]. As illustrated in S1 Fig, in GO AFM images, fewer peaks at a longer distance (9.6 μm) exhibit a bifurcated shape and a slight elevation in height. Conversely, GO-PEI AFM images display an increased number of peaks with lower heights at a shorter sampling distance (2.55 μm). This observation probably serves as additional evidence underscoring the impact of PEI conjugation in the exfoliation of graphene oxide layers. Alternatively, the presence of bifurcated peaks in GO with increased height may stem from the accumulation of graphene oxide layers at different angles or potentially arises from the corrugated structure exhibited by graphene oxide sheets [26], a feature not observed in GO-PEI AFM images.

#### 3.3. Evaluation of produced polyplexes

**Fig 5:**
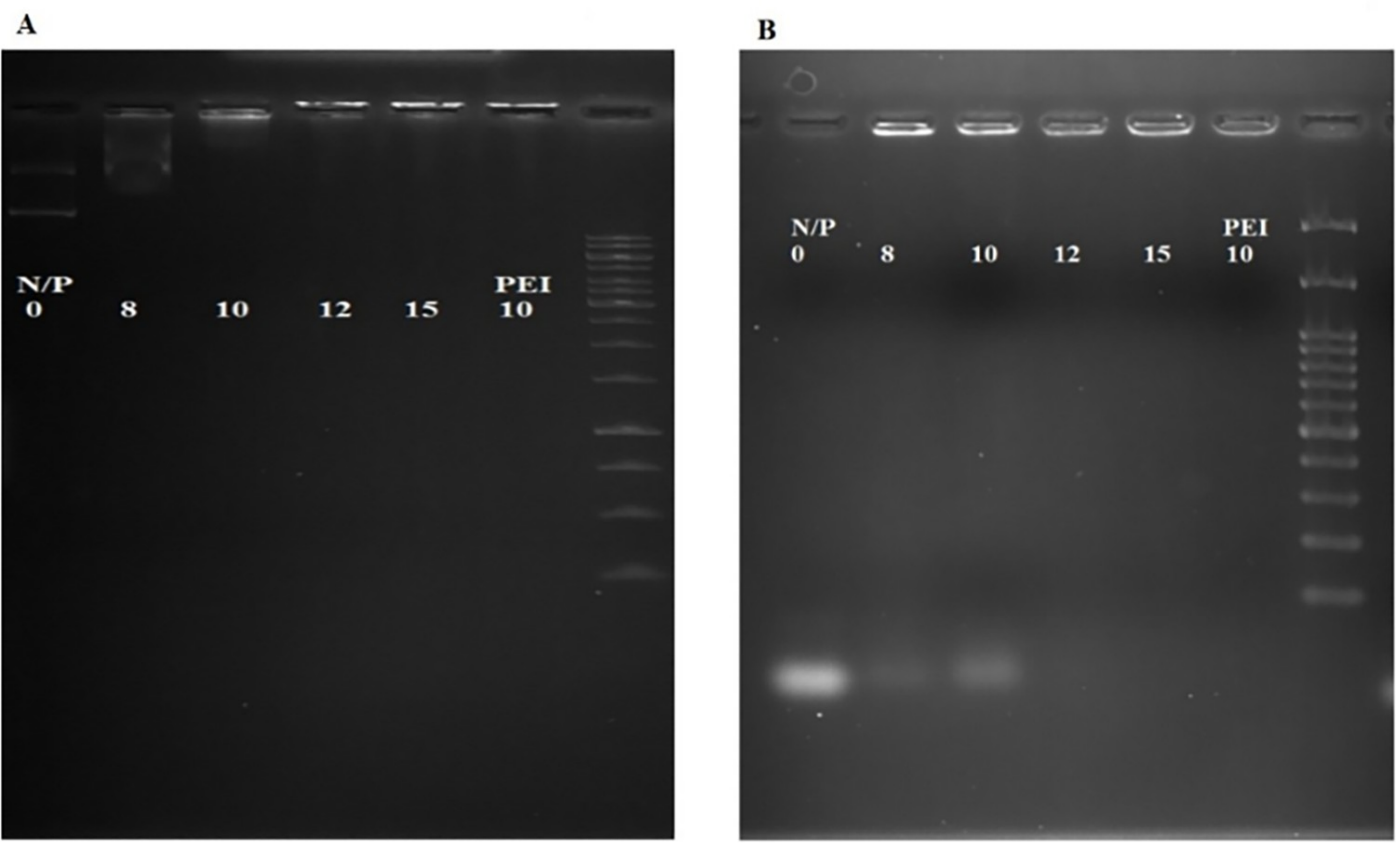
Gel retardation assay. (A) Polyplexes formed with plasmid and GO-PEI. Polyplexes of siRNA and GO-PEI. (B) The amount of utilized DNA and siRNA in all proportions of polyplexes was 1 μg.

**Fig 6:**
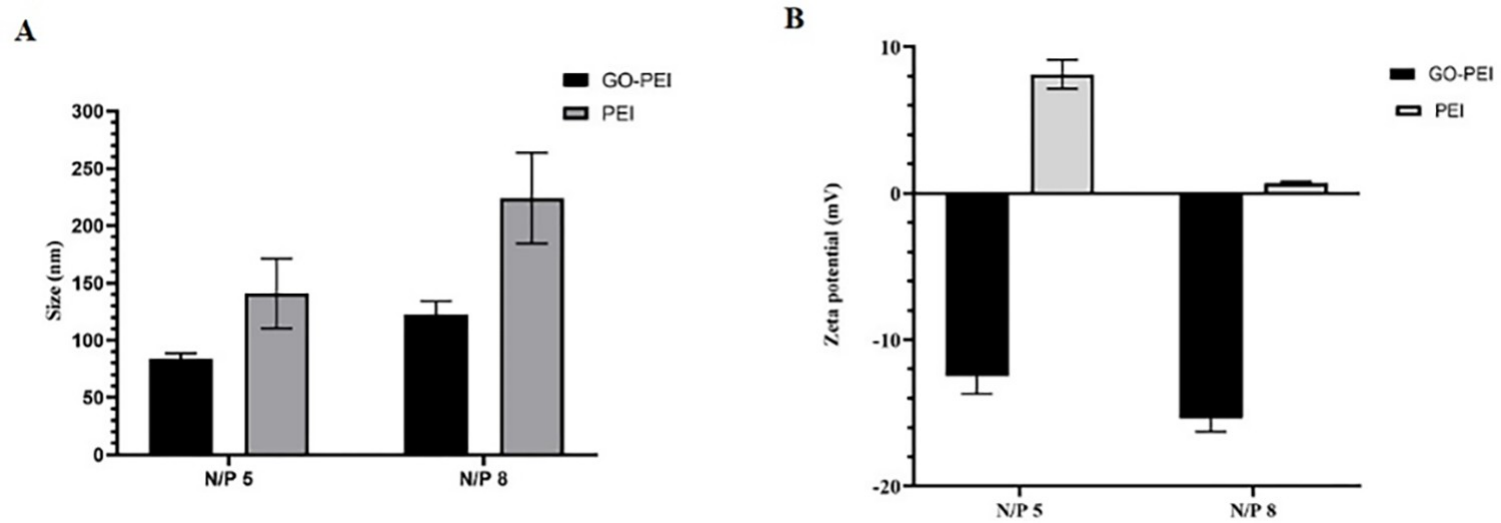
DLS analysis of polyplexes. (A) The hydrodynamic size of polyplexes with N/P 5 and N/P 8. (B) Zeta potential of polyplexes with N/P 5 and N/P8. The data are presented with mean ± SD.

The condensation of DNA by nano-carriers is a prerequisite for transferring genetic materials into the cells. For this, GO-PEI particles and DNA or siRNA were electrostatically bound together at various N/P ratios, ensuring that all formulations contained an equal amount of DNA (1 μg). After 30 minutes of incubation at room temperature, the polyplexes were loaded onto an agarose gel to evaluate the accuracy of their formation, comparing their mobility with bare nucleic acids. The anticipated outcome was a retarded migration of well-condensed polyplexes during electrophoresis, as depicted in Fig 5. As N/P ratios increased, the migration of polyplexes was consistently retarded. Notably, GO-PEI polyplexes with N/P 12 and 15, like PEI polyplexes at N/P 10, remained trapped inside the wells, indicating the efficient condensation of genetic materials with GO-PEI. In addition, the size and zeta potential of polyplexes were assessed by DLS (Fig 6). The zeta potential of polyplexes was negative, likely attributed to DNA loading on nano-carriers and changing their surface charge from positive to negative by inducing conformational changes and exposing the functionally negative groups of graphene at the surface of polyplexes. The hydrodynamic size of GO-PEI polyplexes with N/P 8 was 120 nm on average.

### 3.4. Cell toxicity

**Fig 7:**
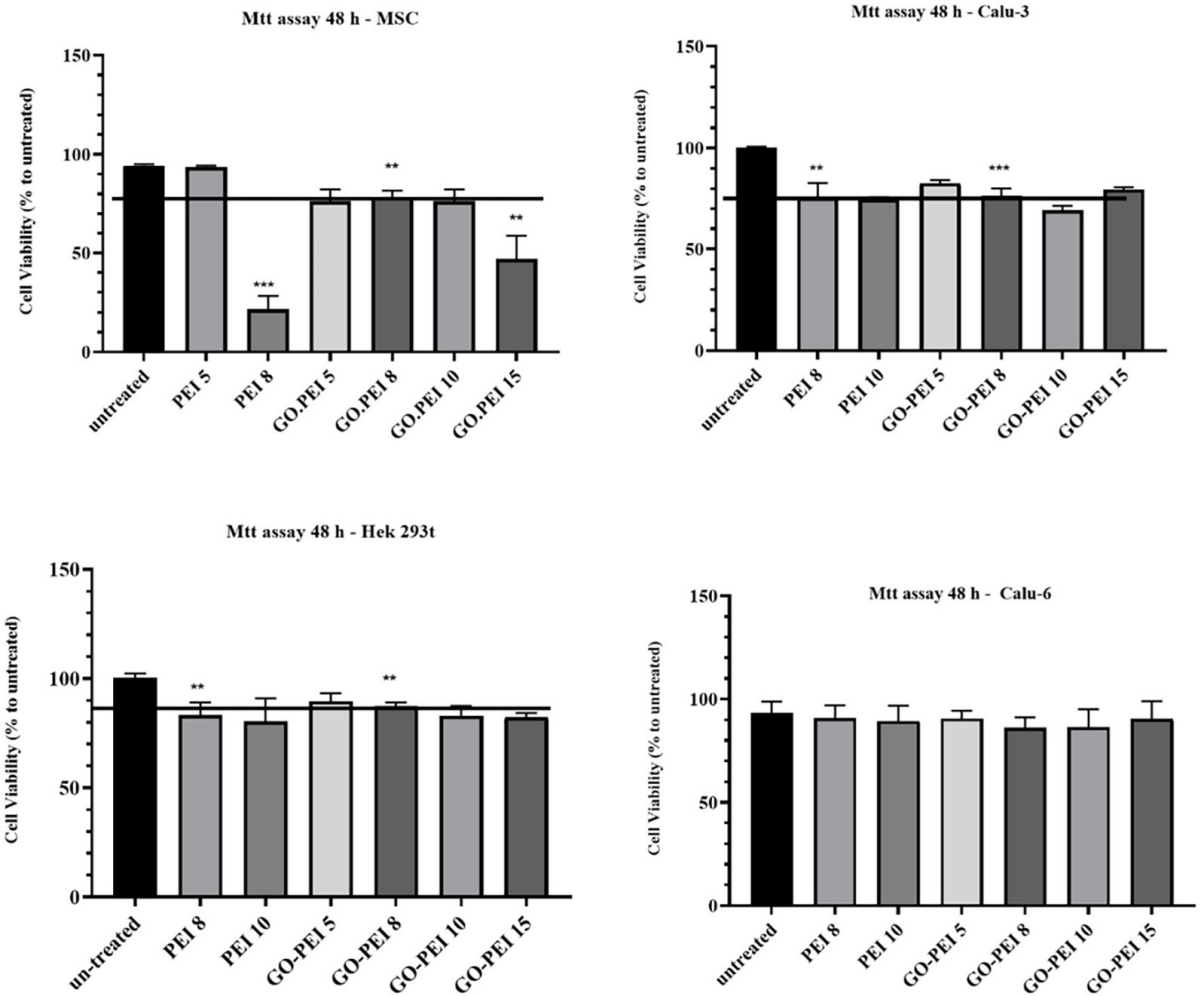
MTT assay graphs. The cell viability affected by GO-PEI and PEI was evaluated by treating cells with identical amounts of nano-carriers used in N/P ratios of polyplexes, excluding nucleic acids, to select the safest N/P ratio for transfection experiments. Therefore, the number of treatments (e.g., PEI 8, GO-PEI 5 etc.) refers to the N/P ratios. Of note, N/P 5, N/P 8, N/P 10, N/P 15 equals 1/8 μg, 2.8 μg, 3.58 μg, 5.4 μg of nano-carriers in these graphs.

The safety of nano-carriers is crucial for their efficient function as transfection reagents. However, the toxicity of each nanomaterial varies depending on the cell type. Therefore, determining the optimal dosage of the nano-carrier for each specific cell type is essential. In this study, an MTT assay was performed to evaluate the toxicity of GO-PEI on the studied cell types. Our MTT assay data indicated the toxicity effects of GO-PEI on the cell viability to some extent, which disappointed us for its potential use in gene therapy approaches. It is worth noting that additional cell toxicity assays are necessary to ascertain whether a nano-carrier is safe to use as a delivery system in vitro or in vivo. In MTT experiments, we treated cells with the identical amounts of nanoparticles, which were utilized in each N/P proportion of polyplexes but without any genetic materials, aiming to understand the precise effect of GO-PEI as a nano-carrier on cell viability. According to the MTT results, GO-PEI reduced the viability of MSCs significantly. This toxicity effect was also evident by observing cells with a microscope 24 hours after transfection. The viability of MSCs was dramatically reduced by increasing the PEI amount from N/P 5 to N/P 8. Consequently, only N/P 5 was employed to transfect MSCs with PEI polyplexes. GO-PEI treatments also induced a significant decrease in the viability of Calu-3 cells. The viability of Calu-6 wasn’t affected significantly by these concentrations of GO-PEI. The toxicity effect of GO-PEI in HEK293T cells was also significant, albeit seemingly trivial, when compared to MSC and Calu-3 cells (Fig 7).

#### 3.5. Transfections

Fluorescent microscopy and flow cytometry were employed to evaluate the efficiency of transfections by tracking the expression of GFP since the utilized plasmid in polyplexes harbored the GFP gene. Initially, we conducted transfections with various N/P ratios of GO-PEI polyplexes on HEK293T cells, a well-known cell type that can be transfected easily. The expression of GFP in these transfections was astonishing when observed via fluorescent microscopy. Furthermore, we performed a transfection experiment with GO-PEI polyplexes containing fluorescent siRNAs against a long non-coding RNA named *LINK-A* as another proof to indicate GO-PEI’s efficiency in gene transfer and successfully releasing it in HEK293T cells. In this experiment, the observation of transfected cells by fluorescent microscopy confirmed the efficient transfection of siRNAs by this nano-carrier. Additionally, the significant reduction in *LINK-A* expression indicated the successful release of siRNAs from GO-PEI carriers and their subsequent binding to their target RNAs (S2 Fig).

**Fig 8:**
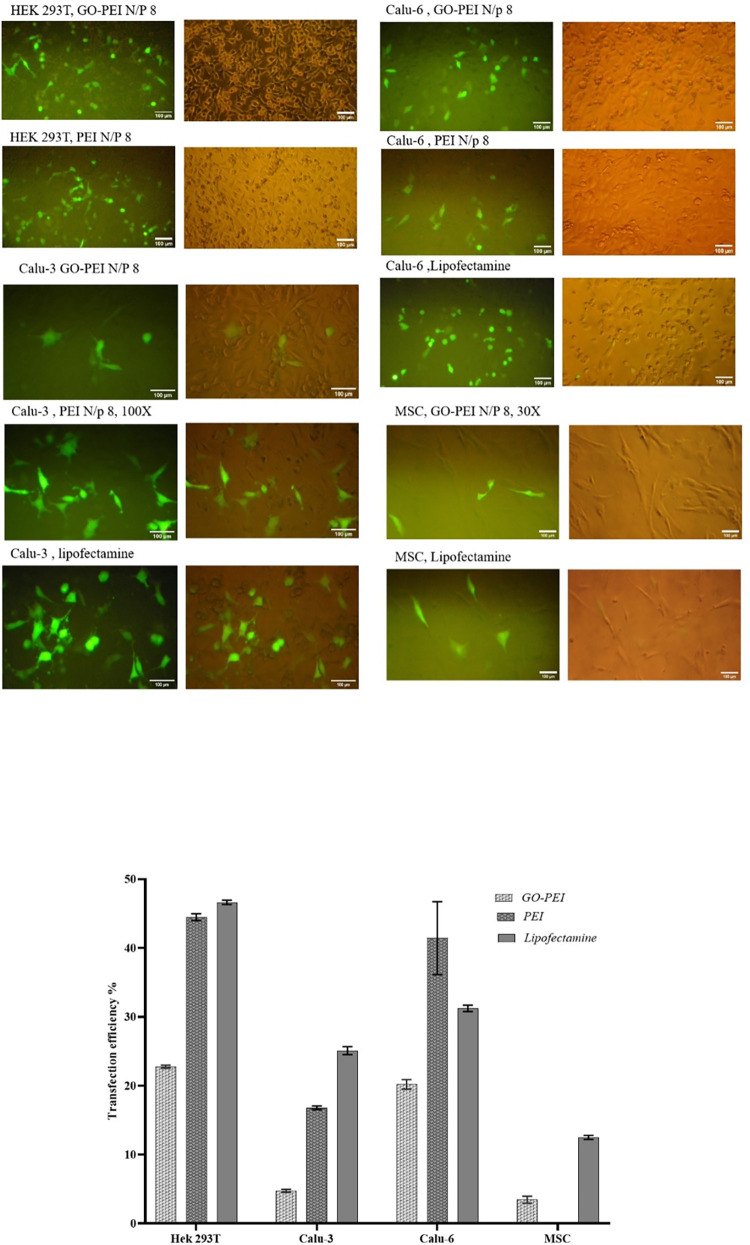
The efficiency of transfections. Fluorescent microscopy images depict transfections in the examined cell types, with scale bars in each image indicating 100 μm. The graphs of quantifying GFP expressions in transfected cells are obtained from a flow cytometry device. The amount of DNA was 1 μg in all transfections, either with nano-carriers or Lipofectamine. The polyplexes with N/P 8 were utilized in all transfection. The graphs are presented with mean ± SEM.

In this study, polyplexes with N/P 8 were reported for transfection of other cell types, and the efficiency of transfection in these cell types was notably lower compared to HEK293T cells. PEI N/P 8 was used as a control positive for all transfections. However, we weren’t able to use PEI in the transfection of primary MSCs because of its massive toxicity effects on this cell type. Thereby, Lipofectamine 2000 was applied as a control positive for comparison with the transfection efficiency of GO-PEI in MSCs. According to the flow cytometry results, the transfection efficiency of GO-PEI was three times lower than that of Lipofectamine 2000 in MSC. Although, we tried several methods to enhance the transfection efficiency of GO-PEI in MSCs, they weren’t successful, probably because of the toxicity of these nano-carriers in this cell type. In all transfection experiments, the pH was set at 7. Transfection efficiency in all cell types with GO-PEI, PEI, or Lipofectamine is compared in Fig 8.

## 4. Discussion

An efficient non-viral delivery system must fulfill several criteria, including good biocompatibility, high loading capacity, and cell penetration ability or transfection efficiency. Graphene oxide seems promising as an efficient gene carrier. In this prospect, we synthesized PEI-coated graphene oxides and examined their efficiency across various cancer cell lines and human BM-MSCs. According to our observations, GO-PEI nano-carriers efficiently transfer GFP-plasmids and siRNAs into HEK293 cells. The efficiency of transfection was not only evident from the fluorescent microscope images, but was also confirmed by the significant down-regulation of the siRNA target gene, indicating efficient delivery of siRNA and its release from GO-PEI carriers. This observation motivated us to investigate the efficiency of our synthesized nano-carriers in transferring plasmids to other cell lines. Various factors, including cell type, confluency during the transfection, media composition, the size or type of gene constructs, the material and size of nano-carriers, and the transfection method, collectively affect the ultimate transfection efficiency [27–29]. PEI compared with GO-PEI demonstrates higher efficiency in transferring genetic materials to most cell types. But the application of bare PEI is not favorable as a transfection reagent because of its extensive cell toxicity. However, the conjugation of graphene oxide with PEI provides an opportunity to merge PEI’s advantages in effectively condensing nucleic acids for efficient transfection with graphene oxide’s high loading capacity and excellent biocompatibility. Moreover, the high surface-to-volume ratio of GO augments its loading capacity for transfection or the simultaneous transfer of drugs and genetic materials. In this case, the cytotoxicity induced by GO-PEI is expected to be lower than that of bare PEI, and MTT assays in our experiments confirmed this. However, our observed transfection efficiencies with GO-PEI were not higher than those transfected with bare PEI. Ren et al. employed PEI 10 kDa in their GO-PEI construction, yielding approximately17% transfection efficiency in HEK293T cells [21], which is lower than our GO-PEI’s transfection rate (22.7%) in the same cell line. This difference might stem from the molecular weight of conjugated PEI on graphene oxide. Nonetheless, they enhanced the transfection efficiency to nearly 35% by adding a nuclear-localized signal peptide to the GO-PEI delivery system [21]. In another study conducted by Teimouri et al., the inclusion of glycine linkers in GO-PEI systems improved the transfection efficiency of plasmids in murine neuroblastoma cells compared to their bare PEI25 kDa control transfection groups [30]. However, the overall transfection efficiencies observed in their study were very low. This lower transfection efficiency, even with PEI 25 kDa, might be attributed to the specific cell type utilized in their experiments.

The average size of our GO-PEI resembles, to some extent, that of Kim et al.’s GO-PEI carriers, ranging between 100 and 200 nm [20]. However, directly comparing transfection efficiencies across different cell types might not be entirely reliable. Nevertheless, we speculate that the improved transfection efficiency observed in their study could potentially be attributed to the lower toxicity of PEI 1.8 kDa in comparison to PEI 25 kDa. Generally, the efficiency of transfection with bare PEI 25 kDa is way better than PEI 1.8 kDa. However, the altered characteristics of the nano-carriers in the conjugated form of GO and PEI could potentially impact the transfection efficiency. Certainly, examining the transfection efficiencies of GO-PEI in identical cell types while varying the weights of PEI in their constructs is invaluable for evaluating this explanation. Wang et al. functionalized graphene oxide with PEI and PEG to transfer siRNAs into an ovarian cancer cell line (SKOV3), then they armed GO-PEI-PEG with folic acids to specifically target ovarian cancer cells with over-expressed folate receptors on their surface. They perfectly indicated that the transfection efficiency of GO-PEI-PEG compared with bare PEI was dramatically lower, but the transfection was incredibly enhanced when the same GO-PEI-PEG was armed with folate acid [31]. The size of their nano-carriers in both conditions was larger than 200 nm, which is larger than the size of our GO-PEI nano-carriers (160nm). Based on these studies, size could not be a reason for the lower transfections in our study. Probably, designing a targeted nano-carrier system for on-target cells could be a reasonable approach to enhance transfection efficiency while minimizing off-target effects. In the present work, we investigated the transfection efficiency of plasmid with our GO-PEI nano-carriers in primary MSCs, which are known for being difficult to transfect. Surprisingly, PEI 25 kDa, even at low N/P ratios, exhibited substantial cytotoxicity toward MSCs. The application of PEI polyplexes resulted in massive cell death in MSCs within 24 hours post-treatment, rendering PEI 25 kDa unsuitable as a positive control for transfection in MSCs. Quantitative analysis of GFP expression revealed that the transfection efficiency of lipofectamine 2000 exceeded that of GO-PEI by threefold in MSCs. Numerous factors might significantly influence the transfection efficiency of MSCs. Despite efforts involving alterations in media, varying starvation times, and employing diverse transfection methods, we did not observe an elevation in the transfection efficiency of GO-PEIs in MSCs. Induced cell toxicity likely restricts this process, potentially interfering with the expression of the transferred gene. MSCs treated with PEI N/P 8 only had 20% viability, whereas MSCs treated with GO-PEI N/P 8 indicated over 70% viability. Therefore, the conjugation of PEI with graphene oxide effectively mitigates the toxicity of PEI in MSCs. Nevertheless, the observed toxicity by GO-PEI in MSC is still excessively high to achieve adequate transfection efficiency. It’s worth mentioning that the transfection efficiency as well as rate of toxicity in primary cells can vary depending on the cell’s sources and donors. For instance, Ramos-Murillo et al. reported 15.3 ± 8.6% transfection efficiency using bPEI 25 kDa in MSCs isolated from Umbilical Cord Wharton’s Jelly. Interestingly, the viability of MSCs treated with PEI in their study was similarly reduced, comparable to our observations with the same concentration of PEI [32]. According to MTT assays in the present work, treatment with GO-PEI reduces the viability of MSC and Calu-3 cells by almost 25%, which are the most declining viability rates among the other studied cell types. The observed toxicity in MSCs by PEI treatment could be related to the sensitivity of primary cells rather than cell lines. Although, the rate of toxicity by the same nano-carrier could vary among different sources of MSCs. The higher cell toxicity of PEI and GO-PEI in Calu-3 cells compared with Calu-6 may be attributed to the presence of the intracellular mucus-containing vesicles in Calu-3 cells, which their fusion with the lysosomal vesicles containing DNA/nano-carrier complexes induce disassociation of polyplexes and leaving the nano-carriers inside the cytoplasm instead of delivering them to the nucleus and consequently causing cell toxicity effects [33]. However, precise cell toxicity and genotoxicity assays are needed to determine the mechanisms behind the observed reduced cell viability by nano-complexes in these cells. Coincidentally, the transfection efficiency of MSC and Calu-3 cells with GO-PEI was the lowest among all transfected cell types. In another study involving MSC transfection, increasing the ratio of Lipofectamine to DNA from 5 to 20 raised the transfection rate from 20% to 40%, respectively, while causing a 30% reduction in cell viability. Accordingly, cell toxicity from the higher amount of lipofectamine did not affect the transfection rate in MSCs adversely [34]. In our study, transfection with higher N/P ratios didn’t improve the transfection efficiency in MSCs, probably because of inducing intensive toxicity by application of greater concentration of GO-PEI in these cells. Another limiting element for a low transfection rate could be the polyplex’s size, which is a significant factor for a successful cellular uptake. It has been proven that polycation-DNA gene carrier systems are usually delivered to the cells by endocytosis or pinocytosis with sizes inferior to 100 nm [35]. Clathrin-mediated endocytosis is a well-known mechanism for internalizing graphene oxides with sizes ranging from 100 nm to 200 nm [36–38]. Particles smaller than 100 nm typically utilize caveolae-mediated endocytosis, while larger particles ranging between 500 and 800 nm primarily rely on macropinocytosis for cellular internalization. Clathrin-mediated endocytosis is virtually the most dominant type of endocytosis across various cell types [39]. Thus, our GO-PEI particles with an average size of 160 nm are well-suited for successful cellular uptake, suggesting that size is unlikely to be the reason for the observed low transfection efficiencies in this study.

Considering specific cell surface molecules for designing targeted delivery systems in MSC appears to be a promising approach for having a successful transfection. Lee et al. remarkably enhanced their transfection efficiency by incorporating hyaluronic acid into their calcium phosphate gene delivery system, leveraging the interaction between hyaluronic acid and CD44 on the surface of MSCs [40]. In another study conducted on MSC by Saraf et al., conjugation of hyaluronic acid and bPEI led to a three-fold elevation in the transfection efficiency of bPEI [41]. These studies clearly indicate the advantage of CD44 and the application of hyaluronic acid for designing MSC-targeted delivery systems. Other reports indicated that priming cells with some compounds can enhance the transfection efficiency in MSC. For instance, dexamethasone, a well-known glucocorticoid, significantly increased the transfection efficiency of MSCs [42]. Probably, dexamethasone plays its enhancing role by reducing the toxicity effects of delivery reagents in the transfection or by decreasing the apoptosis occurrence in transfected cells, ultimately resulting in heightened gene expression of the transferred gene [43]. Nevertheless, priming MSC cells with dexamethasone in our experiments didn’t affect the transfection efficiency. We used 1.2 ×10^−7^ M concentration of dexamethasone for priming, according to Hamann et al. [43]. Although, we had confirmed the safety of this concentration of dexamethasone on cell viability through an MTT assay (S3 Fig).

Chloroquine is another transfection enhancer known to increase the transfection efficiency in most cell types by accumulating in late endosomes and lysosomes [44]; However, priming MSCs with chloroquine didn’t increase the transfection efficiency in our experiments either. As previously noted, the plasmid construct also plays a crucial role in the final transfection rate. For instance, Hamann et al. proved that having a CMV promoter upstream of the GFP or luciferase gene compared with E2F or RSV promoters, causes 10-fold higher gene expression with Lipofectamine 3000 transfection in MSC [45]. In the present work, the utilized promoter for the GFP gene was CMV, and the prospect of plasmid construction seems appropriate for successful transfection.

To the best of our knowledge, this is the first study reporting GO-PEI transfection efficiency in calu-3 and calu-6 cell lines, frequently employed as representations of lung cancer. Calu-3 is a differentiated adenocarcinoma cell type and potentially poses an extra barrier for transfection due to its mucin secretion [46]. Our observations indicated a lower transfection efficiency of GO-PEI in Calu-3 cells compared to the Calu-6 cell line. This disparity might be related to the mucin secretion with Calu-3. Hence, cell type stands as a critical factor in non-viral gene delivery systems, determining the optimal transfection reagent to use [47]. Developing cell-targeted drug delivery systems holds promise for achieving efficient transfection across various cell types.

## 5. Conclusion

Our GO-PEI nano-carriers exhibited successful gene transfer into HEK293T and Calu-6 cells, albeit with lower transfection efficiency compared to Lipofectamine 2000. Nevertheless, this percentage of transfection can be acceptable when the goal is to use the benefits of graphene oxide. Comparing our study with previous research underscores the potential for enhancing transfection efficiency by functionalizing graphene oxide with more biocompatible materials. Moreover, equipment of the delivery system with a specific cell-targeting factor seems crucial for improving transfection efficiency. However, our observations in primary MSCs were not promising, probably influenced by higher toxicity effects.

## Supporting information

S1 Fig Height measurement of nanoparticles using AFM images. (DOCX)

S2 Fig Efficiency of transfection with various N/Ps in HEK 293T cells. (DOCX)

S3 Fig Dexametasone effect in cell viability of MSCs. (DOCX)

S1 Table Oligonucleotide primers used in real-time PCR. (DOCX)

S1 Raw images (PDF)
