# Supplementary material for: In vitro assessments of nanoplexes of polyethylenimine-coated graphene oxide-plasmid through various cancer cell lines and primary mesenchymal stem cells
Source: PLoS One. 2023 Dec 14;18(12):e0295822. doi: 10.1371/journal.pone.0295822 (PMC10720998; doi:10.1371/journal.pone.0295822)
Supplement: S1 Fig — (DOCX) [file pone.0295822.s001.docx]

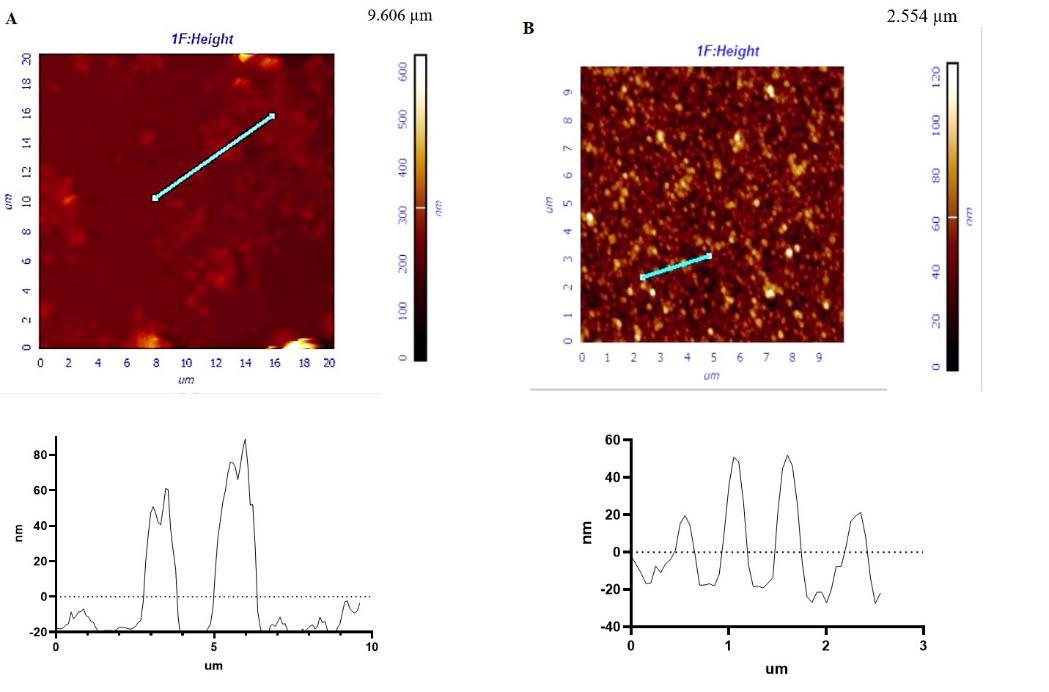


**S1 Fig. Height measurement of nanoparticles using AFM images.** (A) The height measurements indicated the highest peak around 80 nm in graphene oxide, the blue line indicates the sampling length (9.606 µm). (B) Height for GO-PEI particles is around 60 nm, the blue line shows 2.55 µm sampling length. Graphs of height peaks are obtained using Gwyddion software.
