# Supplementary material for: In vitro assessments of nanoplexes of polyethylenimine-coated graphene oxide-plasmid through various cancer cell lines and primary mesenchymal stem cells
Source: PLoS One. 2023 Dec 14;18(12):e0295822. doi: 10.1371/journal.pone.0295822 (PMC10720998; doi:10.1371/journal.pone.0295822)
Supplement: S2 Fig — (DOCX) [file pone.0295822.s002.docx]

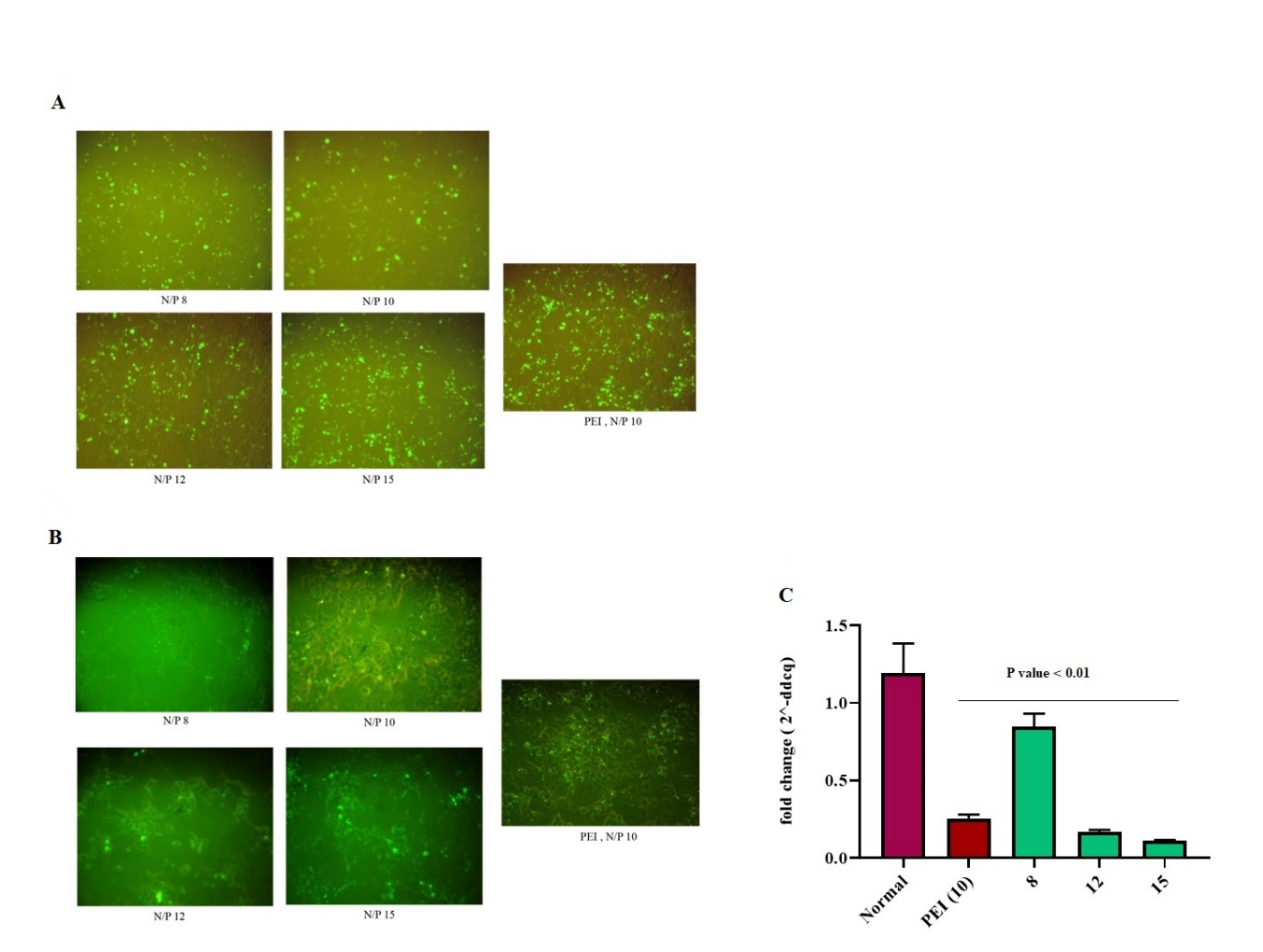


**S2 Fig**. **Efficiency of transfection with various N/Ps in HEK 293T cells.** (A) Transfections of HEK293T cells with GO-PEI and plasmid polyplexes with N/P 8, 10, 12, 15, and PEI and plasmid polyplexes with N/P 10. (B) Transfections of HEK293T cells with GO-PEI and siRNA polyplexes with the same N/P ratios, and PEI and siRNA polyplexes with N/P 8. PEI was used as a control positive for transfections. The images were taken with 10 X magnification of a fluorescent microscopy. (C) qPCR proved the efficiency of transfections by indicating the significant reduction of LINK-A lncRNA, which is the target of utilized siRNAs.
