# Supplementary material for: In vitro assessments of nanoplexes of polyethylenimine-coated graphene oxide-plasmid through various cancer cell lines and primary mesenchymal stem cells
Source: PLoS One. 2023 Dec 14;18(12):e0295822. doi: 10.1371/journal.pone.0295822 (PMC10720998; doi:10.1371/journal.pone.0295822)
Supplement: S3 Fig — (DOCX) [file pone.0295822.s003.docx]

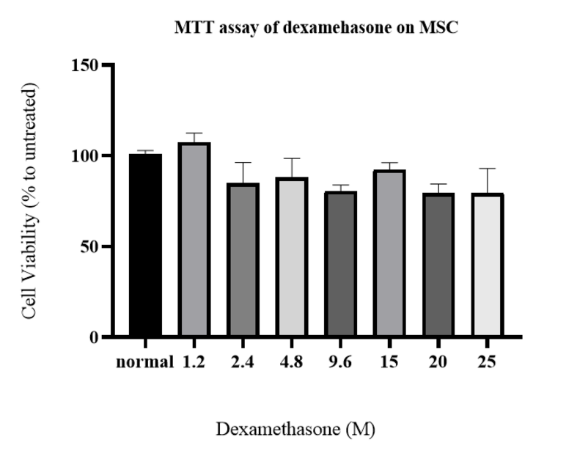


**S3 Fig. Dexametasone effect in cell viability of MSCs.** MTT assay graph indicating effect of various concentrations of dexamethasone on MSC. The data are presented with mean ± SEM.
