# Supplementary material for: In vitro assessments of nanoplexes of polyethylenimine-coated graphene oxide-plasmid through various cancer cell lines and primary mesenchymal stem cells
Source: PLoS One. 2023 Dec 14;18(12):e0295822. doi: 10.1371/journal.pone.0295822 (PMC10720998; doi:10.1371/journal.pone.0295822)
Supplement: S1 Table — (DOCX) [file pone.0295822.s004.docx]

**S1 Table. Oligonucleotide Primers used in Real-time PCR**

| *Link-A* F | ACAGCTCATTTATCCATTTTCCTAC |
| --- | --- |
| *Link-A* R | CAGAGATATACACAACAATTTCATACC |
| *GAPDH* F | CATCAAGAAGGTGAAGCAG |
| *GAPDH* R | GCGTCAAAGGTGGAGGAGTG |
