# Supplementary material for: In vitro assessments of nanoplexes of polyethylenimine-coated graphene oxide-plasmid through various cancer cell lines and primary mesenchymal stem cells
Source: PLoS One. 2023 Dec 14;18(12):e0295822. doi: 10.1371/journal.pone.0295822 (PMC10720998; doi:10.1371/journal.pone.0295822)
Supplement: S1 Raw images — (PDF) [file pone.0295822.s005.pdf]

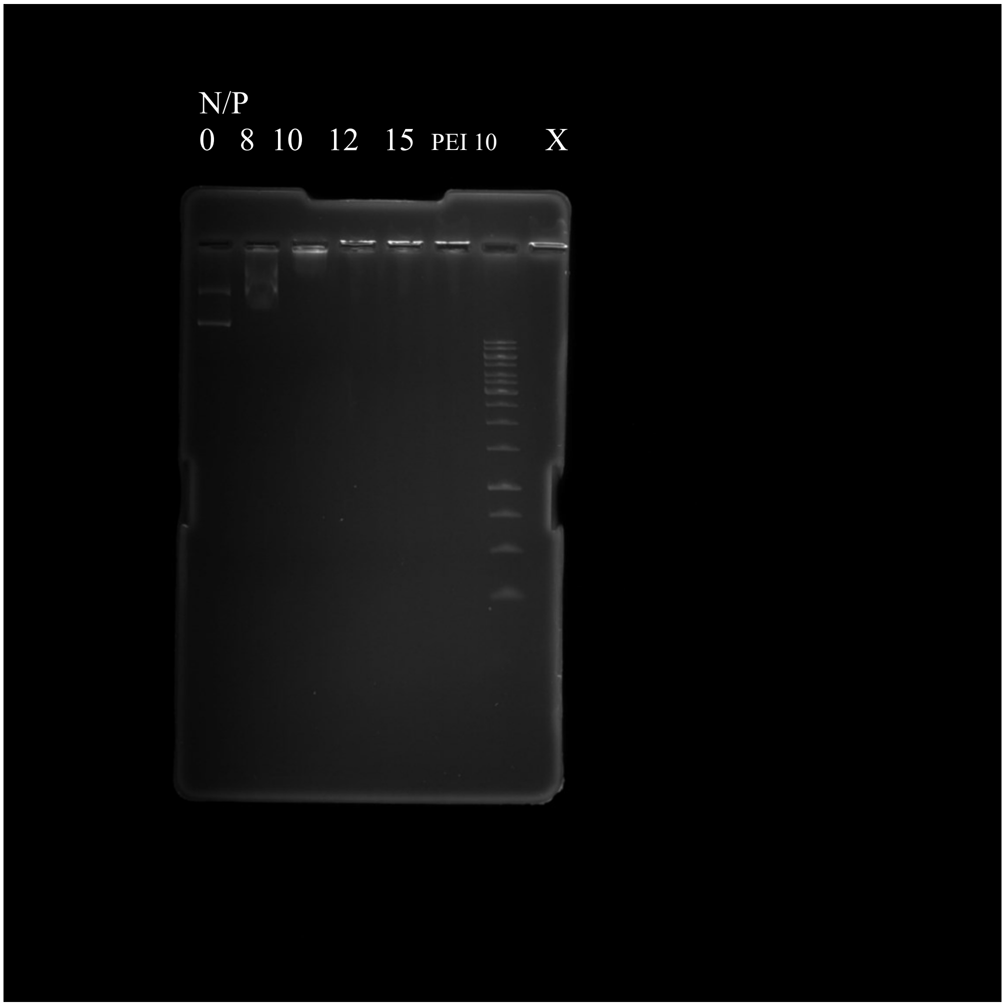

Gel electrophoresis of GO-PEI or PEI polyplexes with plasmid. 1 μg plasmid was used in all N/P ratios. Wells from left to right were loaded with Plamid (N/P 0, 1μg plasmid without any nano-carrier), N/P 8, N/P 10/ N/P 12, N/P 15, PEI 10, and DNA marker respectively. X was loaded with another polyplex. The heaviest and lightest band of the DNA marker is 10 kbp and 250 bp, respectively.

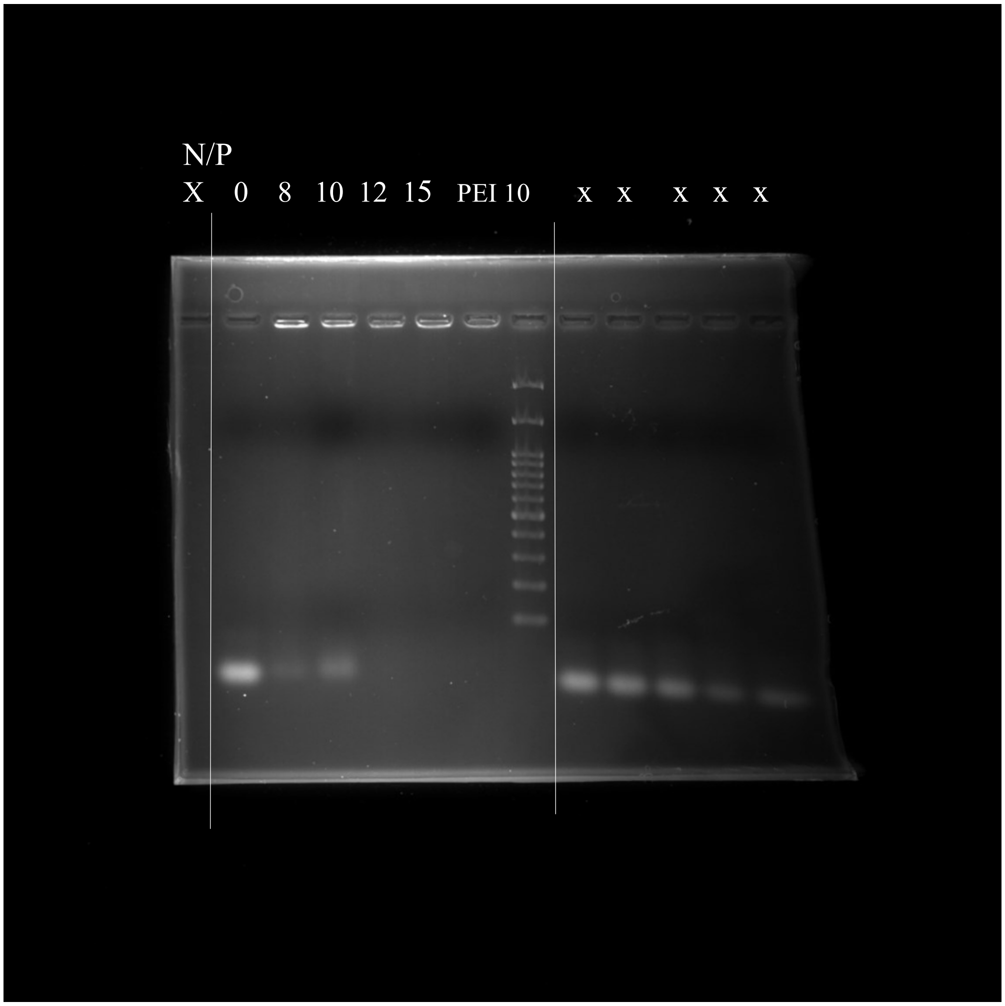

Gel electrophoresis of polyplexes of GO-PEI or PEI with siRNA. Wells from left to right were loaded with x, N/P 0 (1 µg siRNA without any nano-carrier, N/P 8, N/P 10, N/P 12, N/P 15, PEI 10, DNA marker (The upper and lower bands are respectively 3000 and 100 bp), respectively. Wells indicated with X are blank or loaded with other samples irrelevant to this work. The white lines indicate the place where are cropped in the manuscript image (Fig 5).
